# Angiotensin II is required to induce exaggerated salt sensitivity in Dahl rats exposed to maternal separation

**DOI:** 10.14814/phy2.12408

**Published:** 2015-05-21

**Authors:** Analia S Loria, David M Pollock, Jennifer S Pollock

**Affiliations:** 1 Department of Pharmacology and Nutritional Sciences, University of KentuckyLexington, Kentucky; 2 Department of Medicine, Georgia Regents UniversityAugusta, Georgia; 3 Cardio-Renal Physiology & Medicine, Division of Nephrology, Department of Medicine, University of Alabama at BirminghamBirmingham, Alabama

**Keywords:** Acute behavioral stress, angiotensin II, Dahl salt-sensitive rat, maternal separation

## Abstract

We previously reported that maternal separation, rat model of early life stress, enhances pressor responses to acute and chronic stressors. The aims of this study were to determine whether Dahl salt-sensitive (DS) rats subjected to maternal separation (MatSep-DS) as compared to normally reared DS (Ctl-DS) rats show exaggerated blood pressure responses to acute behavioral stressors, such as restraint stress or air jet stress (AJS), or, hypertensive stimuli including chronic high-salt diet (4% NaCl) and angiotensin II (AngII) infusion (200 ng/Kg/min) during 1 week. MatSep was performed in male DS rats for 3 h/day from postnatal days 2–14. At 8 weeks of age, rats were implanted with telemetry transmitters and allowed to recover. Mean arterial pressure (MAP) was not different between MatSep-DS and Ctl-DS rats at baseline (120 ± 2 mmHg vs. 118 ± 1 mmHg, *n* = 4–8). Blood pressure responses during AJS and restraint stress were not different between MatSep-DS and Ctl-DS at 3 min. However, blood pressure recovery from AJS was significantly impaired in MatSep-DS rats compared to Ctl-DS rats (*P* < 0.05). 3-h stress-induced similar responses in MatSep and Ctl-DS rats. Chronic blood pressure responses to AngII infusion in rats fed a high-salt diet displayed enhanced MAP in MatSep-DS when compared with Ctl-DS rats (167 ± 5 mmHg vs. 152 ± 2 mmHg, p_interaction_ <0.05). However, MAP increased similarly in both groups in response to AngII infusion or high-salt diet separately. Renal parameters such as proteinuria, urine flow rate, and urine electrolytes were not different between groups in response to each treatment. In summary, salt sensitivity induces exaggerated blood pressor responses only in presence of AngII due to early life stress.

## Introduction

Exposure to low socioeconomic status, parental loss, sexual or physical abuse, and/or parental dysfunction during childhood, defined as early life stress (ELS), is associated with permanent changes in the adult health and behavior status (Alastalo et al. 2012; Low et al. 2009). For instance, exposure to ELS greatly increases the risk of developing chronic adult diseases, such as hypertension, ischemic heart disease, obesity, anxiety, and depression (Thomas et al. 2008).

Maternal separation (MatSep) during early postnatal life is a model of chronic behavioral stress utilized by our laboratory and others to mimic ELS-induced phenotypes (Loria et al. 2010a,b, 2011, 2013b). We previously reported that MatSep does not induce baseline changes in metabolic and cardiovascular parameters in adult rats (Loria et al. 2010b); however, we have shown that MatSep significant blood pressure sensitivity to acute behavioral stress as well as chronic angiotensin II (AngII) infusion in Wistar-Kyoto (WKY) rats (D'Angelo et al. 2010; Loria et al. 2010a,b).

Dahl salt-sensitive (DS) rats are utilized as a model to evaluate parameters of genetic salt-sensitive hypertension. Prehypertensive DS rats are more sensitive to incremental changes in dietary sodium content compared to Dahl salt-resistant counterparts (Mattson et al. 2004; Sterzel et al. 1988). Furthermore, studies from our laboratory have reported that DS rats subjected to acute behavioral stress have an exacerbated pressor response as well as impaired blood pressure recovery (D'Angelo et al. 2010). An acute stress-induced rise in blood pressure results from autonomic and neuroendocrine-mediated changes in conductance and peripheral vascular resistance (Herd 1991; D'Angelo et al. 2006; Ulrich-Lai et al. 2006). It has been shown that plasma endothelin (ET-1) is one of the vasoactive peptides released in response to acute and chronic stress (Treiber et al. 2000). Even among healthy persons, peripheral ET-1–mediated endothelial dysfunction can last for about 90 min after completion of laboratory-induced behavioral stress (Spieker et al. 2002). The processes by which stress may modulate ET-1 release not fully known; yet several clinical studies provide support for the role of ET-1 in stress-induced cardiac ischemia (Wilbert-Lampen et al. 2008; Fernandez et al. 2010).

As hyperreactivity to stressors increases future risk for cardiovascular disease complications (McEwen and Seeman 1999; Chida and Steptoe 2010), we sought to investigate whether the acquired exaggerated blood pressure reactivity induced by MatSep would be evident in a genetic prehypertensive model such as the DS rat contributing to an increased risk to impair blood pressure control. Therefore, the aims of this study were to test the hypothesis that DS rats subjected to maternal separation (MatSep-DS) as compared to normally reared DS rats induces further exaggerated blood pressure responses to: (i) acute behavioral stressors, including air jet stress (AJS) and restraint-induced stress, or, (ii) prohypertensive stimuli achieved by chronic high-salt diet feeding and/or chronic angiotensin II (AngII) infusion.

## Methods

### MatSep protocol

MatSep was performed as previously described using offspring from DS (MatSep-DS) in-house breeders originally purchased from Charles River Laboratories (Loria et al. 2010b). All experiments were conducted in accordance with the National Institutes of Health Guide for the Care and Use of Laboratory Animals, approved and monitored by the Georgia Regents University Institutional Animal Care and Use Committee. From postnatal days 2 to 14, approximately half of the male offspring were transferred to a clean cage in an incubator (30 ± 1°C) for 3 h for 12 consecutive days. MatSep groups consisted of rats from at least three different litters. Nonhandled counterparts that were not disturbed from their mother served as controls (Ctl-DS). Weaning was performed at postnatal day 28 and experiments were conducted starting at 11 weeks of age.

### Radiotelemetry

Rats were implanted with telemetry transmitters at 8 weeks of age (Data Sciences, Inc., St. Paul, MN) as previously described (Loria et al. 2010b). Mean arterial pressure (MAP) and heart rate (HR) were continuously recorded throughout the study using the Dataquest ART Acquisition program (Data Sciences International, St. Paul, MN). At the age of 11 weeks, rats were subjected to different hypertensive stimuli such as behavioral stress, increased dietary sodium, or chronic infusion of AngII.

### Acute behavioral stress paradigms

We evaluated blood pressure responses to two paradigms of acute behavioral stress: (i) air jet stress in a 3-min period followed by a poststress 20-min period, and (ii) restraint stress in a 3-h period followed by a 2-h poststress period. Rats were moved from the original cage to tubular plexiglass restrainers (D'Angelo et al. 2010). Air jet stress protocol (AJS) was performed as previously reported (D'Angelo et al. 2005). Briefly, all rats were acclimatized (acclimated) to the restrainer tube prior to the experimental day. The day of the determination, MAP, HR, and locomotor activity were monitored in animals for 1 h in their home cages using Dataquest ART Acquisition program (Data Sciences, Inc.), and then placed in tubular plexiglas restrainers for 15 min while continually undergoing telemetry recording. The change in MAP (delta MAP) was determined as the peak of MAP after moving the rat in the restraint minus the average MAP at baseline (of the 3 h prior to moving) in the original cage. AJS consisted of pulses (2 sec every 10 sec × 3 min) of compressed air (15 Ib/in^2^) directed at the rat's forehead. Rats were allowed to recover for 10 days following the AJS protocol. Rats were then placed in the restraint tubes for a 3-h period. MAP and HR were continuously recorded for 3 h. Changes in MAP and HR were expressed as area under the curve during stress and recovery time. After behavioral tests, rats were returned to their home cages.

The total pressor response during 3-min or 3-h stress period was determined by the equation: ∑ ((AJS pressure- pre-AJS pressures) × 0.067 min)). Data are expressed as the area under the curve (AUC; mmHg × 3 min or mmHg × 3 h) as previously described.

### Chronic treatment protocols

All groups of rats were fed and maintained on a normal salt diet (NSD, 0.4% NaCl, Teklad, Harlan Laboratories, Madison, WI). Rats were fed a high-salt diet for 7 days (HSD, 4% NaCl, Teklad, Harlan Laboratories), or infused with AngII (AngII, 200 ng/kg/min (Moreno et al. 2003), Phoenix Pharmaceuticals, Burlingame, CA), or a combination of both. Osmotic mini-pumps containing AngII (model 2002; Alzet, Palo Alto, CA) were implanted subcutaneously under isoflurane anesthesia and sterile conditions according to the manufacturer's instructions as described previously in a subgroup of rats (Loria et al. 2010b).

### Urine and plasma measurements

Rats were placed in metabolic cages for determination of water and food intake as well as urine excretion. After 2 days of acclimation, 24-h urine collections were taken at baseline and at day 7 of treatment. At the end of the experiment, blood was obtained from a direct puncture into the abdominal aorta under anesthesia. The plasma obtained was used to measure levels of ET-1 (Quantikine ELISA kit, R&D, Minneapolis, MN).

Creatinine concentration in plasma and urine was measured by the kinetic Jaffé method using a 10% picric acid solution in NaOH and reading after 15 min in a microplate set for dual wavelengths at 490 nm (read) and 620 nm (reference). Creatinine clearance (Ccr) was calculated using the following formula: Ccr (mL/min) = (Urine Creatinine/Plasma Creatinine) × urine flow rate.

Urinary protein excretion (UprotV) was determined by a colorimetric assay using the Quick Start Bradford Dye Reagent 1X (Bio-Rad, Hercules, CA). After 5 min of incubation, sample reading was performed in a microplate at 595 nm.

Urinary electrolytes Na+ and K+ were measured with a flame photometer (Cole-Parmer, Vernon Hills, IL) in 24-h urine samples and data was expressed in mmol/day.

### Statistical analysis

All data are expressed as mean ± SEM. Unpaired Student's t-test was performed to make comparisons between MatSep-MS and Ctl-control measurements at different time points. Telemetry data are presented as the mean ± SEM at 24 h. For statistical analysis, 144 time points for every 24 h (10 sec average value every 10 min) for each rat were computed. Comparisons between MatSep-DS and Ctl-DS at the end points were made by two-way repeated measures ANOVA followed by a Bonferroni post hoc test. A value of *P* < 0.05 was considered statistically significant. All statistical analyses were conducted using GraphPad Prism 5.01 (GraphPad Software, Inc., La Jolla, CA).

## Results

### Acute behavioral stress and cardiovascular reactivity

**Figure 1:**
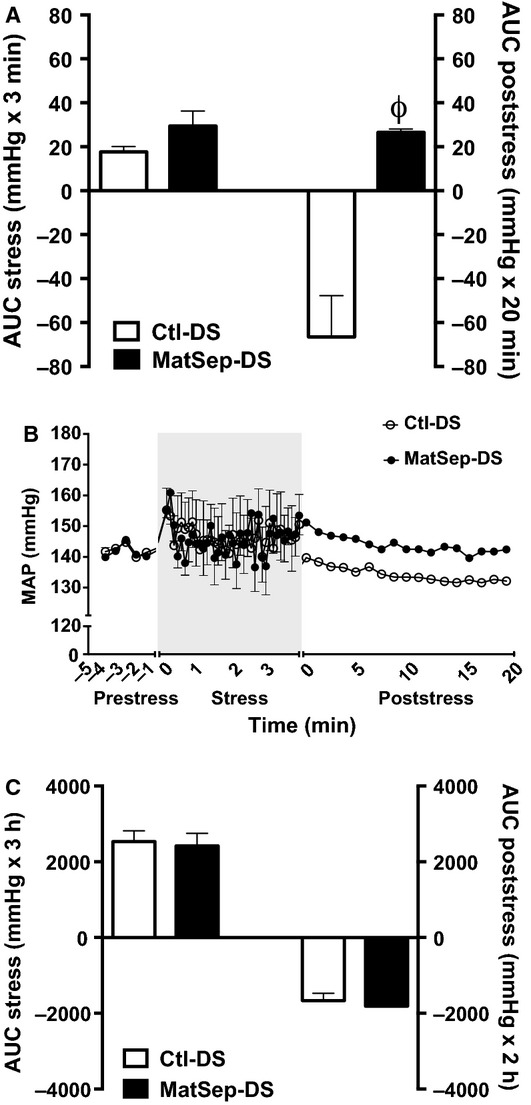
Behavioral stress-induced changes in MAP during stress and recovery from stress in MatSep-DS and Ctl-DS in response to (A) 3-min AJS (B) Representative blood pressure trace in response to acute behavioral stress and (C) 3-h restraint stress. The total pressor response during 3-min or 3-h stress period was determined by the equation: ∑ ((AJS pressure- pre-AJS pressures) × 0.067 min)). ^ϕ^*P* < 0.05 versus Ctl-DS.

At baseline, MAP was not different between MatSep-DS and Ctl-DS rats (120 ± 2 mmHg vs. 118 ± 1 mmHg). MatSep-DS rats showed a greater change in MAP in response to the transfer from the home cage to restraint tubes (16 ± 5 mmHg vs. 36 ± 6 mmHg, *P* < 0.05). Total area under the curve (AUC) of the pressor response to AJS (Fig.1A) was not different between MatSep-DS (6.6 ± 0.7 mmHg × 3 min) and Ctl-DS rats (7.6 ± 1.3 mmHg × 3 min). Blood pressure recovery from AJS was significantly impaired in the MatSep-DS rats compared to the Ctl-DS rats (Fig.1A). Figure1B show the blood pressure trace during prestress, stress, and poststress periods. MatSep-DS and Ctl-DS rats had similar pressor response and recovery of the blood pressure to restraint stress (Fig.1C).

### Chronic blood pressure responses to HSD and AngII treatment

**Figure 2:**
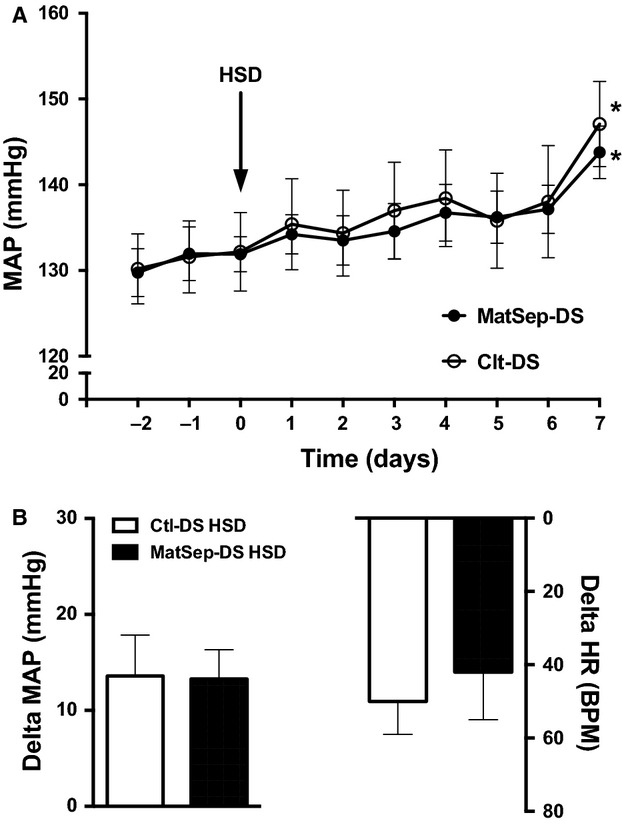
Effect of a 7-day HSD treatment. (A) MAP trace, (B) delta MAP and HR. *P < 0.05 versus baseline same group. Delta = difference in MAP or HR values between day 7 of treatment and baseline.

**Figure 3:**
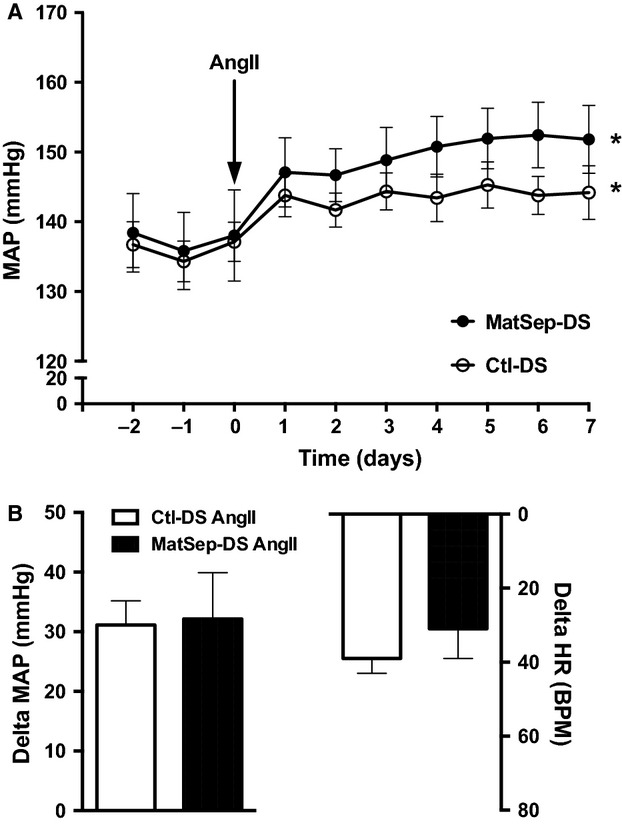
Effect of a 7-day AngII treatment. (A) MAP trace, (B) delta MAP and HR. *P < 0.05 versus baseline same group. Delta = difference in MAP or HR values between day 7 of treatment and baseline.

**Figure 4:**
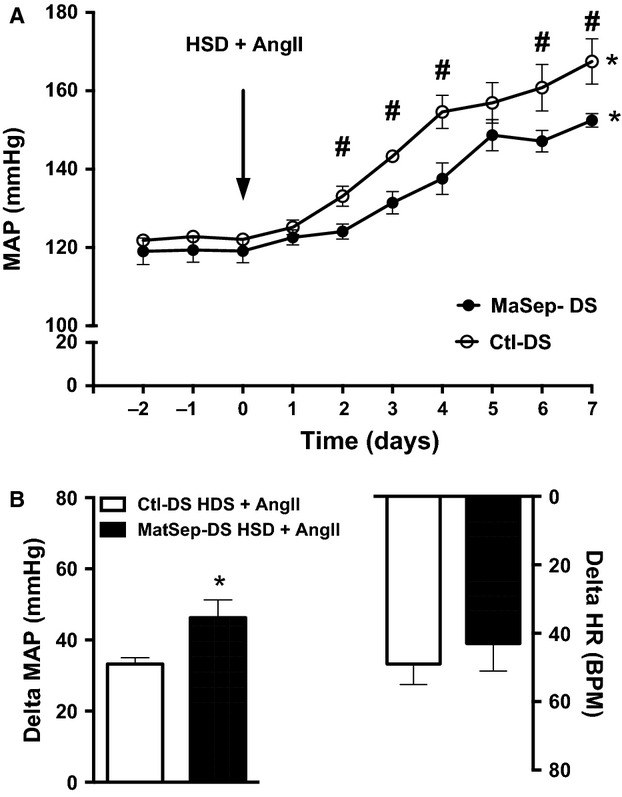
Effect of a 7-day HSD + AngII treatment. (A) MAP trace, (B) delta MAP and HR. *P < 0.05 versus baseline; ^#^*P* < 0.05 versus Ctl-DS; *P < 0.05 versus baseline same group. Delta = difference in MAP or HR values between day 7 of treatment and baseline.

One week on HSD induced a ~15 mmHg rise in mean arterial pressure. Also, heart rate increased similarly between MatSep-DS and Ctl-DS rats (Fig.2A). Chronic AngII infusion exerted a similar increase in MAP between groups as well (Fig.3A). However, MatSep-DS rats displayed an exacerbated blood pressure response to HSD feeding in the presence of a chronic AngII infusion (Fig.4A). HSD (from 385 ± 10 to 33523 ± 14 bpm in MatSep-DS and from 392 ± 24 to 342 ± 17 bpm in Ctl-DS, *P* < 0.05) and AngII + HSD (from 419 ± 7 to 376 ± 8 bpm in MatSep-DS and from 418 ± 7 to 369 ± 7 bpm in Ctl-DS, *P* < 0.05) treatments reduced HR in MatSep-DS and DS compared with NSD fed rats; however, AngII treatment did not have a significant effect on HR (from 402 ± 3 to 387 ± 21 bpm in MatSep-DS and from 407 ± 8 to 380 ± 17 bpm in Ctl-DS) (Figs.2B, 3B and 4B).

### Metabolic parameters and plasma ET-1 levels

Body weight, food and water intake, urine flow, proteinuria, and electrolytes were not different between MatSep-DS and Ctl-DS at baseline (Table1). In addition, no differences were seen in C_cr_ at baseline (Ctl-DS: 1.17 ± 0.08 mL/min DS; MatSep-DS: 1.02 ± 0.07 mL/min).

**Table 1:** Body weight and metabolic parameters on a NSD or after 7 days on a HSD, AngII or AngII + HSD

	NSD *N* = 10		HSD *N* = 8		AngII *N* = 4		HSD + AngII *N* = 4	
	Ctl-DS	MatSep-DS	Ctl-DS	MatSep-DS	Ctl-DS	MatSep-DS	Ctl-DS	MatSep-DS
Body weight (g)	310 ± 3	317 ± 4	321 ± 2	325 ± 5	300 ± 6	305 ± 5	318 ± 4	328 ± 3
Food intake (g)	23 ± 1	22 ± 1	22 ± 1	22 ± 1	21 ± 1	21 ± 2	22 ± 2	20 ± 2
Water intake (mL)	30 ± 1	30 ± 1	61 ± 5*	65 ± 5*	56 ± 7*	46 ± 4*	64 ± 3*	69 ± 7*
Urine flow rate (mL/day)	16 ± 1	17 ± 1	52 ± 4*	47 ± 2*	47 ± 9*	38 ± 3*	57 ± 3*	45 ± 4*

NSD, normal salt diet; HSD, high-salt diet; AngII, Angiotensin II; Ctl-DS, controls; MatSep-DS, maternal separation.

* *P* < 0.05 versus NSD.

**Figure 5:**
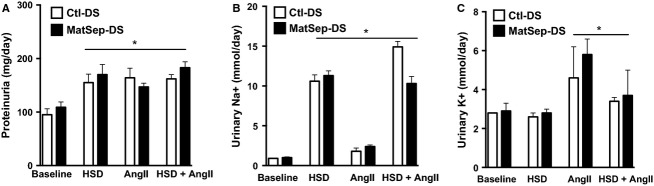
Effect of HSD, AngII or their combination in (A) proteinuria, (B) Sodium excretion, and (C) Potasium excretion. *P < 0.05 versus baseline same group.

Water intake, urine flow, proteinuria, and electrolytes were increased in a similar fashion in both Ctl-DS and MatSep-DS rats in response to HSD, AngII or their combination (Table1, Fig.5A, B). Potassium excretion was increased in AngII-infused rats only (Fig.5C).

At baseline, plasma ET-1 levels were not different between MatSep-DS and Ctl-DS rats (Table2). After 1 week on a HSD, AngII, or HSD + AngII, plasma ET-1 levels were not different between MatSep-DS and Ctl-DS (Table2).

**Table 2:** Plasma ET-1 levels in NSD or after 7 days of HSD, AngII or AngII + HSD

	NSD *N* = 10		HSD *N* = 8		AngII *N* = 4		HSD + AngII *N* = 4	
	Ctl-DS	MatSep-DS	Control	MatSep-DS	Control	MatSep-DS	Control	MatSep-DS
ET-1 (pg/mL)	0.41 ± 0.05	0.49 ± 0.09	0.56 ± 0.02	0.53 ± 0.01	0.51 ± 0.09	0.54 ± 0.01	0.48 ± 0.05	0.63 ± 0.15

NSD, normal salt diet; HSD, high-salt diet; AngII, Angiotensin II; Ctl, control; MatSep, maternal separation; DS, dahl salt-sensitive.

## Discussion

This study shows that ELS induces a modest increase in the sensitivity to prohypertensive stimuli in DS rats, a strain that has been shown to be susceptible to behavioral and diet stressors (Francis et al. 2002; Chida and Steptoe 2010; D'Angelo et al. 2010). We observed an impaired blood pressure recovery in response to acute stress in MatSep-DS rats that may contribute to exaggerated cardiovascular risk. Chronic stressors such as HSD or AngII induced a similar response in both experimental groups in the cardiovascular parameters. However, chronic HSD feeding induced an exaggerated blood pressure response in MatSep-DS rats only in the presence of AngII infusion.

It has been shown that a greater reactivity to stress or delayed poststress recovery in response to acute behavioral stressors predicts future cardiovascular disease risk (Gerin and Pickering 1995; Light et al. 1999; Matthews et al. 2004; Chida and Steptoe 2010). Thus, psychological factors affecting the cardiovascular system increase future risk for cardiovascular disease, such as stroke, hypertension, and preclinical atherosclerosis (Dong et al. 2004; Low et al. 2009; Chida and Steptoe 2010). In addition, an acute stress-induced rise in blood pressure results from autonomic and neuroendocrine-mediated changes in cardiac contractility and peripheral vascular resistance. We found that MatSep-DS rats display a moderate increase in sensitivity to acute behavioral stress. Although changes in blood pressure during stress were not significant due to ELS, increased pressor response to sudden changes in the housing manner (from cage to restraint) was evident in MatSep-DS rats. This finding indicates that MatSep-DS rats are most likely susceptible to behavioral stress, although we were not able to quantify the response using a standardized method. While this study did not examine directly the role of the vasculature, the impaired blood pressure recovery suggests that a blunted endothelial function may contribute to impair the reduced blood pressure levels during poststress period. Taken together, these data show that MatSep-DS rats display a minor effect on acute blood pressure response and a substantial impairment in blood pressure recovery from behavioral stress, which indicates a potential increased risk to develop cardiovascular disease.

When rats were subjected to restraint stress challenge (3 h), we did not observe any difference in the stress-induced blood pressure elevation nor recovery of blood pressure. Therefore our data suggest that MatSep impairs the endothelial-dependent factors released acutely to buffer sudden and critical changes in blood pressure. These factors may not be required in response to restraint stress.

Chronic treatments with HSD or AngII individually induced similar responses in MatSep-DS and Ctl-DS rats. However, only the combination of HSD and AngII induced exaggerated hypertension in MatSep-DS rats, providing evidence that AngII is enhancing the effects of salt sensitivity on blood pressure levels. In terms of cardiac output, HR was reduced after 1 week on HSD or AngII + HSD. In addition, the AngII-treated group showed a nonsignificant but noticeable reduction in HR as well. Because of these results, we suggest that the baroreflex function does not seem to be compromised in MatSep-DS rats.

To further investigate the contributing factors involved in DS rats blood pressure regulation, we analyzed the levels of ET-1. It has been shown that DS rats develop an ET-1-dependent hypertension. Previous studies from our laboratory using DS rats show that acute behavioral stress triggers an elevated plasma ET-1 level that increases blood pressure (Loria et al. 2010a). Chronically, several studies have shown that ET-A receptor blockade can attenuate the salt-induced hypertension in DS rats (Barton et al. 1998; Kassab et al. 1998; Zicha et al. 2012). Furthermore, animal models also have shown that ET-1 plays a key role mediating salt-dependent hypertension in DS rats (Kohan et al. 2011) Mattson et al. 2004; Ikeda et al. 1999). Although our results show that plasma ET-1 was not increased in MatSep-DS rats, there was no direct measurement of production of ET-1 in various tissues. Therefore, the study of ET-1 receptor expression in different tissues may reveal differences that could be important for the changes observed in blood pressure.

At the metabolic level, we found similar body weight, food and water intake and urinary electrolytes excretion among groups. The outcomes due to AngII, HSD or their combination on water intake, diuresis, and natriuresis in Ctl-DS rats were not significantly exacerbated in MatSep-DS rats compared to controls. Although MatSep-DS rats show signs of sodium retention, the increase in blood pressure observed in response to HSD + AngII does not correlate with significant changes in water and sodium homeostasis between groups; however, small or undetectable changes in sodium balance may contribute with the chronic elevation in blood pressure. Essential hypertensive patients display widely different levels of blood pressure but are usually in sodium balance, nevertheless, both clinical and experimental studies have shown that there is an alteration in the pressure–natriuresis curves that contributes to the development of hypertension (Navar 1997). An optimal renal function is required to prevent excessive sodium and volume depletion and maintain a normal pressure–natriuresis relationship. The salt-sensitive hypertension in DS rats is linked to renal dysfunction (Dahl and Heine 1975). Specifically in DS rats, loss of glomerular filtration rate (GFR) autoregulation contributes with the rightward shift in pressure–natriuresis curve toward higher perfusion pressures (Roman 1986). While treatments with angiotensin-converting enzyme (ACE) inhibitors or Ang II receptor type 1 (AT1) receptor antagonists fail to prevent the development of hypertension (Kodama et al. 1997; Otsuka et al. 1998, 2000; Sugimoto et al. 1998), several studies have shown that ACE inhibitors or AT1 receptor antagonists ameliorated progressive sclerotic and proliferative glomerular changes (Otsuka et al. 1998, 2000), and increased the life expectancy of DS rats (Kodama et al. 1997; Kim et al. 2001). However, the specific mechanisms responsible for the renoprotective effects of AT1 receptor blockade remain unclear. We have reported previously that reduced GFR and signs of glomerular damage and proteinuria support one of the proposed mechanisms by which MatSep impairs chronic blood pressure response in adult WKY rats (Loria et al. 2013a). Thus, we propose that a more detailed study of the renal hemodynamics and the salt-AngII interactions may provide further insights in the mechanisms underlying enhanced elevated blood pressure in MatSep-DS rats.

One limitation of this study is the length of the chronic exposure to salt and AngII. We used 7 days period given the extremely high blood pressure developed in rats on a HSD in presence of AngII. However, in a separate study, we fed rats on a HSD for a total of 3 weeks. Although rats reached the expected high blood pressure levels, we did not find significant differences between groups (data not shown).

In summary, the use of a prehypertensive strain revealed the expected responses to behavioral stress, high salt or AngII; however, early life stress did not modify these outcomes significantly. Thus, we suggest that a prehypertensive genetic background does not exacerbate the responses to different environmental insults when rats were subjected to maternal separation. Nevertheless, a possible cross talk between the AngII and salt signaling pathways appears to enhance the hypertensive response in MatSep-DS rats, which requires further examination in subsequent studies.
